# Role of Palm Oil Vitamin E in Preventing Pre-eclampsia: A Secondary Analysis of a Randomized Clinical Trial Following ISSHP Reclassification

**DOI:** 10.3389/fmed.2020.596405

**Published:** 2021-01-21

**Authors:** Nurul Afzan Aminuddin, Rosnah Sutan, Zaleha Abdullah Mahdy

**Affiliations:** ^1^Community Health Department, Faculty of Medicine, Universiti Kebangsaan Malaysia Medical Center, Kuala Lumpur, Malaysia; ^2^Obstetrics and Gynaecology Department, Faculty of Medicine, Universiti Kebangsaan Malaysia Medical Center, Kuala Lumpur, Malaysia

**Keywords:** preeclampsia prevention, antioxidant, palm oil vitamin E, tocotrienol-rich fraction, pregnancy induced hypertension

## Abstract

**Background:** Preeclampsia is a significant cause of maternal and perinatal mortality worldwide. Oxidative stress plays a key role in its pathophysiology, hence antioxidants such as tocotrienol may be preventive against preeclampsia. In 2018, the ISSHP revised the definition of preeclampsia. In accordance with the new definition, we report a secondary data analysis from a clinical trial comparing palm oil vitamin E in the form of tocotrienol-rich fraction (TRF) against placebo, in preventing preeclampsia.

**Method:** A randomized double-blind controlled trial was conducted in 2002–2005 to assess the benefits of TRF in preeclampsia prevention. A total of 299 primigravidae were recruited. The intervention group was supplemented with TRF 100 mg daily in super-olein capsules, whereas the placebo group was prescribed super-olein capsules without TRF, beginning from 12 to 16 gestational weeks until delivery. The primary outcome measure was incidence of preeclampsia.

**Results:** The total incidence of pregnancy induced hypertension (PIH) was 5%, whereas the incidence of preeclampsia was 2.3%. The odds of developing PIH (adjusted OR 0.254; 95% CI: 0.07–0.93; p–value 0.038) and preeclampsia (adjusted OR 0.030; 95% CI: 0.001–0.65; *p*-value 0.025) were significantly lower in the TRF arm compared to the placebo arm.

**Conclusion:** Antenatal supplementation with palm oil vitamin E in the form of TRF is associated with significant reductions in the incidence of preeclampsia and PIH in a single urban tertiary hospital. Palm oil vitamin E deserves further scrutiny as a potential public health preventive measure against preeclampsia and PIH.

## Introduction

Hypertensive disorders of pregnancy (HDP) was reported as one of the main risk factors leading to bad outcome of pregnancies (1). Urbanization has driven the human population to practice unhealthy lifestyles that lead to increasing trends of non-communicable diseases (2).

The prevalence of HDP is 5–10% (3) with preeclampsia affecting 3–5% of pregnancies (1). Preeclampsia was traditionally diagnosed by the combined presentation of high blood pressure and proteinuria. New definitions include maternal organ dysfunction, such as renal insufficiency, liver involvement, neurological or hematological complications, and uteroplacental dysfunction as evidenced by fetal growth restriction (FGR).

In year 2001 the International Society for the Study of Hypertension in Pregnancy (ISSHP) updated the HDP classification (4), followed by another revision in 2014 (5) and subsequently in 2018 (6). The revised classification reflected new perspectives and understanding that influenced management of this disease.

Approximately 287,000 preventable maternal deaths occur annually worldwide and HDP is listed as one of the major contributing factors (7). Other than early diagnosis and prompt appropriate treatment (8, 9), screening and prevention is vital in order to reduce morbidity and mortality especially in low resource settings where accessibility to healthcare is inadequate (10, 11). Currently calcium and aspirin (6, 12–14) have established, albeit small, roles in the primary prevention of preeclampsia.

Oxidative stress in the placenta leads to systemic maternal inflammatory response causing maternal vascular endothelial dysfunction in preeclampsia (15, 16). The significant evidence of oxidative stress playing a key role in the development of preeclampsia directed to the hypothesis that antioxidant supplementation may have a role in preventing the disease. The potential of antioxidants such as vitamins C, E, selenium and lycopene (17) in the prevention of conditions associated with oxidative stress like preeclampsia, is supported by evidence-based studies (18–21).

Vitamin E, specifically tocotrienol, is a powerful antioxidant that has been shown to reduce oxidative stress and prevent propagation of free radical reactions in female reproductive health (22, 23). The two major forms of vitamin E are tocopherol and tocotrienol (24). Tocotrienol is 40–60 times more potent as an antioxidant, with superior anti-inflammatory properties compared to tocopherol (24).

Palm oil Vitamin E in the form of tocotrienol-rich fraction (TRF) has been shown to increase the antioxidant levels of older adults (22). However, there is limited clinical trials on the effects of palm oil vitamin E in women of reproductive age (25).

We have published the outcome of our randomized controlled trial (RCT) on the role of tocotrienol in prevention of preeclampsia (25) using the old definition (4). With recent revision of the classification of HDP, we decided to review our results and add more perspectives into the data analyses.

## Methods

### Design Overview

The present study used secondary data from a previous randomized double-blind controlled trial (Grant Number: 06-02-02-0136) (25), where the intervention group was supplemented with palm oil vitamin E (TRF 100 mg daily in super olein) capsules and the placebo group was prescribed super olein capsules, beginning from 12 to 16 gestational weeks until delivery. The project was carried out at a single tertiary hospital in an urban setting in Malaysia from June 2002 to March 2005 by recruiting primigravidae from the antenatal clinic as stated in the published article (25). Sample size calculation, inclusion and exclusion criteria, method of double blinding and randomization, and other details have been described previously (25).

The secondary data covered a total of 299 samples in four different excel files, comprising the subjects' sociodemographic profiles, obstetric profiles, and admission and delivery details. The validity and completeness were verified during data collection and analysis by the previous research team.

All 299 samples were included in this secondary data analysis. Preeclampsia was redefined according to the new definition by ISSHP (6). Fetal growth (clinical and sonographic) data and laboratory findings such as full blood count, renal profile, and liver function test were reviewed for criteria fulfilling the updated diagnosis of preeclampsia.

### Primary Outcome

The incidence of preeclampsia is the primary outcome measure in this secondary data analysis. The revised definition of preeclampsia according to the ISSHP in 2018 (6) was used in this study, and applied to secondary data from the previously published RCT. The incidence of preeclampsia was re-determined in fulfillment of the new ISSHP criteria. In 2001, besides a clinical definition of preeclampsia, the ISSHP suggested a research definition that must include significant proteinuria (4), and this was adopted as the definition of preeclampsia in our original study (25). However, in the revised classification and diagnosis in 2018, this research definition was no longer included in the ISSHP recommendations (6).

The birthweight data was recoded into status of fetal growth restriction either yes or no (according to WHO growth chart and weeks of gestation at delivery). Urine creatinine was recoded into more than 90 and <90 μmol/l for determination of status of renal function. Platelet count was recoded into <150 and more than 150/μl for presence of thrombocytopenia. The diagnosis of preeclampsia was subsequently re-determined based on the recoded criteria.

### Secondary Outcomes

This is defined as the incidence of (1) pregnancy induced hypertension (PIH, defined as gestational hypertension and preeclampsia); and (2) maternal and fetal outcomes, including onset of labor (either spontaneous or induced), mode of delivery (either vaginal or cesarean section), gestation at delivery and baby's birth weight.

### Independent Variables

The independent variables in this study comprise sociodemographic characteristics (age, race, occupation, household income), gestation at recruitment, TRF supplement status and other nutritional supplements.

Age was defined in years at recruitment based on date of birth. Race was categorized and recoded into Malay, Chinese, Indian and others according to major ethnicities in Malaysia. Occupation was categorized and recoded into health care worker group (doctor, nurse, medical laboratory technician etc.); educator group (teacher, lecturer, tutor, etc.); “others” (defined as occupations other than medical and educator groups); and, unemployed (defined as home maker or student with no income nor contributing toward household income). Household income was categorized into three groups based on monthly family income (1) more than MYR 5,000; (2) within MYR 5,000–3,000; and (3) < MYR 3,000.

Gestation at recruitment was defined as period of gestation (in weeks) at recruitment, either based on the date of the last menstrual period or on ultrasonography.

TRF was defined as Yes if the participant received TRF (intervention group) or No if the participant received placebo (control group). Other nutritional supplements was defined as supplements either prescribed by medical personnel [such as folic acid, ferrous fumarate, vitamin C, tablet Obimin^TM^ (Unam Pharmaceuticals), tablet Iberet^TM^ (Abbott Pharmaceuticals)] or other nutritional supplements taken by the participant on their own accord.

### Data Analysis

Data from the previous RCT was recorded in Excel spreadsheets and coded according to operational definition of study variables. The data was then transferred to IBM Statistical Package for Social Sciences (SPSS) version 21 software for further statistical analysis. Univariate descriptive analysis was performed by using frequency (*n*) and percentage (%) for categorical data, and using mean and standard deviation (SD) for normally distributed continuous data, or median and interquartile range for data that is not normally distributed. Data exploration was performed to assess the normality of quantitative data distribution by using Kolmogorov–Smirnov test, mean, median, mode, skewness and kurtosis values.

The bivariate analysis was performed using chi-square test for categorical variables to compare participant/maternal characteristics including sociodemographic profile, gestation at recruitment, other supplements taken by participants, and pregnancy outcome such as the occurrence of HDP, preeclampsia, onset of labor, mode of delivery (vaginal or cesarean section), preterm delivery, and low birth weight between the intervention group and the control group.

Multivariate analysis was performed by using multiple logistic regression to control for confounders in order to assess the role of TRF in preventing PIH and/or preeclampsia. Intention-to-treat analysis was not performed as data was unavailable from 20 participants who did not complete the study.

## Results

The total number of participants who completed the RCT was 299, with 151 participants in the TRF arm and 148 in the control arm. The incidence of PIH was 5% and the incidence of preeclampsia 2.3% according to the latest definitions and classification published by ISSHP (6). The incidence of PIH was no different compared to the primary data analysis (25). After re-categorization according to the new classification, the incidence of preeclampsia remained the same in the TRF arm, but increased in the placebo arm (from 3.4 to 4.1%), compared to the primary analysis. Total incidence of preeclampsia was therefore increased.

The differences in relative risk (RR) of PIH and preeclampsia (TRF vs. control arm) between the old and new classification/definition is shown in Table 1. The risk of developing either PIH or preeclampsia is lower in the TRF arm compared to the control arm. The differences in the risks of developing preeclampsia and PIH between the two arms were not statistically significant in bivariate analysis, although the *p-*value in the case of RR for preeclampsia approaches significance (*p* = 0.052) following ISSHP reclassification of preeclampsia as shown in Table 1.

**Table 1:** The RR of PIH and preeclampsia in the TRF arm compared to the control arm, when defined according to the latest ISSHP classification of HDP (6) compared to the older classification (4).

**Outcome**	**RR**	**95% Confidence Interval**	***p***
**PIH (gestational hypertension** **+** **preeclampsia)**			
Previous classification 2001	0.36	0.12–1.09	0.058
ISSHP reclassification 2018	0.36	0.12–1.09	0.058
**Pre-eclampsia**			
Previous definition 2001	0.20	0.02–1.66	0.094
Revised ISSHP definition of pre-eclampsia 2018	0.16	0.02–1.34	0.052

Comparison of maternal characteristics between the TRF and control arms is shown in Table 2, whereas comparison of pregnancy outcome between the two arms is shown in Table 3. There are no significant differences in secondary outcome measures between the two arms.

**Table 2:** Maternal characteristics of participants in the TRF arm compared to the control arm.

**Characteristic**	**TRF (*****N*****:151)**		**Control (*****N*****:148)**		***P***
	***n***	**(%)**	***n***	**(%)**	
Age	Median; 27 (IQR: 4)		Median;27 (IQR:5)		0.9351^*a*^
Maternal weight	Median; 53 (IQR:14)		Median; 53.4 (IQR:12.72)		0.346^*a*^
**Advanced maternal age**					0.770
Less than 35	143	94.7%	139	93.9%	
More than 35	8	5.3%	9	6.1%	
**Race**					
Malay	112	74.2%	111	75.0%	0.436
Chinese	26	17.2%	25	16.9%	
Indian	12	7.9%	8	5.4%	
Others	1	0.7%	4	2.7%	
**Occupation**					0.017
Medical personnel	54	35.8%	40	27.0%	
Educator	10	6.6%	15	10.1%	
Others	62	41.1%	81	54.7%	
Unemployed	25	16.6%	12	8.1%	
**Household income**					0.026
More than MYR 5,000	9	6.0%	1	0.7%	
MYR 3,003–5,000	42	27.8%	51	34.5%	
Less than MYR 3,000	100	66.2%	96	64.9%	
**Gestation at recruitment**					0.064
1st trimester	29	19.2%	17	11.5%	
2nd trimester	122	80.8%	131	88.5%	
**Others supplement**					0.787
Yes	106	(70.2%)	106	71.6%	
No	45	(29.8%)	42	28.4%	

a *Mann-Whitney U Test/ Wilcoxon Rank Sum*.

**Table 3:** Outcome of pregnancy in the TRF arm compared to the control arm.

**Characteristic**	**TRF (*****N*****:151)**		**Control (*****N*****:148)**		***p***
	***n***	**(%)**	***n***	**(%)**	
**PIH**					0.058
Yes	4	2.6%	11	7.4%	
No	147	97.4%	137	92.6%	
**Pre-eclampsia**					0.052
Yes	1	0.7%	6	4.1%	
No	150	99.3%	142	95.9%	
**Onset of labor**					0.603
Spontaneous	104	68.9%	106	71.6%	
Induced	47	31.1%	42	28.4%	
**Mode of delivery**					
Vaginal	112	74.2%	117	79.1%	0.319
Cesarean section	39	25.8%	31	20.9%	
**Preterm delivery**					0.256
Yes	17	11.3%	11	7.4%	
No	134	88.7%	137	92.6%	
**Low birth weight**					0.241
Yes	17	11.6%	11	7.6%	
No	129	88.4%	134	92.4%	

The risk of disease, absolute risk difference (ARD), RR reduction, and number needed to treat (NNT) between the two arms, preeclampsia and PIH, are shown in Table 4. TRF is seen to have significant negative associations with the incidence of PIH and preeclampsia after control of other confounders such as age, maternal weight, race, occupation, household income and gestational age in multivariate logistic regression analysis. The odds of developing PIH and preeclampsia were lower in the TRF arm compared to the control arm. Factors associated with the incidence of PIH and preeclampsia are presented in Tables 5,6. The magnitudes of reduction in the incidence of PIH and preeclampsia with TRF supplementation are 64 and 83% respectively.

**Table 4:** Risk, ARD, RR reduction and NNT in the TRF arm compared to the control arm for both preeclampsia (PE) and PIH.

**Intervention**	**PE**	**Not developed PE**	**Total**	**Risk**
Placebo	6	142	148	6/148 = 0.041
TRF	1	150	151	1/151 = 0.007
ARD		Risk in placebo - Risk in TRF		0.041–0.007 = 0.034
RR reduction		$\frac{Risk\;in\;placebo\;-\;Risk\;in\;TRF}{Risk\;in\;placebo}$		0.829
NNT		1/ARD		1/0.034 = 29
**Intervention**	**PIH**	**Not developed PIH**	**Total**	**Risk**
Placebo	11	137	148	11/148 = 0.074
Vitamin E (TRF)	4	147	151	4/151 = 0.027
ARD		Risk in placebo - Risk in TRF		0.074–0.027 = 0.047
RR reduction		$\frac{Risk\;in\;placebo\;-\;Risk\;in\;TRF}{Risk\;in\;placebo}$		0.635
NNT		1/ARD		1/0.047 = 21

*ARD, absolute risk difference; RR, Relative risk; NNT, number needed to treat*.

**Table 5:** Factors associated with PIH.

**Variables**	**Crude OR^**a**^**	**95% CI**	***p***	**Adj. OR^**b**^**	**95% CI**	***p***
Age	1.00	(0.99; 1.01)	0.961	NS		NS
Maternal weight	1.04	(1.007;1.076)	0.019	1.05	(1.01;1.09)	**0.01**
**Race**			0.391	NS		NS
Malay	1.52	(0.331; 6.94)	0.591			
Indian	0.000	(0.00; 0.00)	0.999			
Others	0.000	(0.00; 0.00)	0.998			
Chinese	1.000					
**Occupation**			0.255	NS		NS
Medical personnel	0.38	(0.052; 2.806)	0.343			
Educator	2.39	(0.369; 15.437)	0.361			
Others	1.03	(0.211; 5.103)	0.964			
Unemployed	1.00					
**Household income**			0.559	NS		NS
More than MYR 5,000	2.61	(0.294; 23.186)	0.389			
MYR 3,003–5,000	1.621	(0.546; 4.814)	0.385			
Less than MYR 3,000	1.000					
**Gestation at recruitment**				NS		NS
2nd trimester	2.636	(0.338; 20.55)	0.290			
1st trimester	1.000					
**TRF**						
Yes	0.339	(0.105;1.090)	0.054	0.254	(0.07; 0.93)	**0.038**
No (placebo)	1.000			1.000		
**Other supplement**						
No	1.136	(0.35; 3.67)	0.830	NS		NS
Yes	1.000					

a *Crude odd ratio using simple logistic regression*.

b *Adjusted odd ratio (multiple logistic regression using Backward likelihood method, Hosmer and Lemenshow test p-value 0.568, Nagelkerke R^2^ = 11.3%); 1.000 = reference*.

*The p values in bold indicate statistically significant differences*.

**Table 6:** Factors associated with preeclampsia.

**Variable**	**Crude OR^**a**^**	**95% CI**	***p***	**Adj. OR^**b**^**	**95% CI**	***p***
Age	0.999	(0.978; 1.021)	0.926	NS		NS
Maternal weight	1.058	(1.014; 1.104)	**0.009**	1.095	(1.030; 1.165)	0.004
**Race**			0.721	NS		NS
Malay	1.382	(0.163; 11.741)	0.767			
Indian	0.000	0.000	0.998			
Others	0.000	0.000	0.999			
Chinese	1.000					
**Occupation**			0.414	NS		NS
Medical personnel	0.783	0.069; 8.900	0.843			
Educator	3.130	0.268; 36.524	0.363			
Others	0.511	0.045; 5.790	0.587			
Unemployed	1.000					
**Household income**			0.465			**0.048**
More than MYR 5,000	5.333	(0.540; 52.72)	0.152	63.575	(2.287; 1,767.603)	**0.014**
MYR 3,003–5,000	1.055	(0.190; 5.866)	0.951	1.338	(0.219; 8.154)	0.752
Less than MYR 3,000	1.000			1.000		
**Gestation at recruitment**	1.314	(0.621;2.781)	0.475	NS		NS
**TRF**						
Yes	0.16	(0.02; 1.33)	0.042	0.030	(0.001; 0.65)	**0.025**
No(placebo)	1.000			1.000		
**Others supplement**						
No	1.857	(0.407; 8.476)	0.424	NS		NS
Yes	1.000					

a *Crude odd ratio using simple logistic regression*.

b *Adjusted odd ratio (multiple logistic regression using Backward likelihood method, Hosmer and Lemenshow test p-value 0.544, Nagelkerke R^2^ =26.5%); 1.000 = reference. NS, Not Significant*.

*The p values in bold indicate statistically significant differences*.

## Discussion

The reclassification of HDP by ISSHP signifies an important change in the perception of the disease and acknowledges the wider spectrum of disease manifestation compared to previous schools of thought. In line with this the outcome of studies in recent years should be reviewed to take into consideration this new rationale in definition and classification.

This secondary analysis of a double blind RCT on TRF vs. placebo in prevention of PIH and preeclampsia uncovered interesting differences when preeclampsia was redefined according to the latest ISSHP classification of HDP. We found an increase in the incidence of preeclampsia to 2.3%, compared to 2.0% when calculated on the basis of the older definition by ISSHP in 2001 (4). The incidence of PIH remained 5%, as in the primary analysis. In view of the presence of significant confounders, multivariate analysis was performed, demonstrating that the odds of PIH and preeclampsia were significantly lower among women who were prescribed palm oil vitamin E intervention in the form of TRF compared to those who received placebo.

Bivariate analysis only summarizes the relationship between two variables which are one predictor variable and one outcome variable, whereas multivariate analysis simultaneously analyses more than one predictors and have an ability to control the confounding effects for preeclampsia such as maternal weight and socioeconomic status (26). In multivariate analysis after controlling other variables (such as maternal age, weight, race, occupation, household income and intake of other supplements), TRF significantly reduced the risk of PIH and preeclampsia. The adjusted odds of developing PIH is 0.254 [(95% CI; 0.07–0.93), *p*-value: 0.038], which means the risk is lower in the TRF arm, compared to the control arm. Similarly, the adjusted odds of developing preeclampsia is 0.030 [(95% CI: 0.001; 0.65), *p*-value: 0.025] in the TRF arm compared to the placebo arm.

The calculated absolute risk reduction was 4.7% for PIH and 3.4% for preeclampsia. Thus, TRF may reduce the risk of a patient developing PIH by 4.7% and preeclampsia by 3.4%. The NNT were 21 for PIH and 29 for preeclampsia. This implies that, 21 pregnant women should be treated prophylactically with TRF in order to prevent one occurrence of PIH, and 29 in the case of preeclampsia. These findings support public health measures on primary prevention of PIH and preeclampsia using palm oil vitamin E in the form of TRF, in addition to the currently available prophylaxis of aspirin and calcium.

Another important, albeit unsurprising finding, is that maternal weight was significantly associated with PIH, hence healthy lifestyle and eating habits should continue to be promoted and emphasized. Interestingly, household income of more than RM 5,000/month is significantly associated with preeclampsia. However, this finding should be interpreted cautiously and should not be generalized to other populations (especially in rural settings), since this study was conducted in a single urban tertiary hospital.

The protective effect of a strong antioxidant agent against preeclampsia supports the belief that oxidative stress plays a vital role in the pathophysiology of the disease. Therefore, TRF with its substantial antioxidant capacity (16) has good potential for use as one of the preventive measures against preeclampsia, especially in mothers with low baseline levels of antioxidant (27).

To the best of our knowledge, this is the only study so far where vitamin E, in the form of TRF from palm oil, demonstrated protection against PIH and preeclampsia. Other studies do not support the role of vitamin E in preventing either preeclampsia or PIH (18, 27–34). However, the other studies used vitamin E in the form of the much weaker antioxidant tocopherol, either as a monotherapy or in combination with vitamin C.

The safety and efficacy of tocotrienols have been clearly demonstrated in various research and reviews, both in human and animal studies (35–38).

In summary, palm oil vitamin E in the form of TRF 100 mg daily prescribed from before 16 weeks gestation appears to have potential impact in reducing the risk of developing preeclampsia and PIH among primigravidae.

## Limitations

This study was performed in a single urban tertiary hospital in Malaysia, involving only primigravidae. The numbers were too small to conduct a sub-analysis on early-onset vs. late-onset preeclampsia.

## Future Studies

Future research with a larger sample that covers a variety of populations will shed more light on this prophylaxis against preeclampsia. Very importantly, the impact of early onset preeclampsia should be looked into.

## Conclusion

Antenatal supplementation with palm oil vitamin E in the form of TRF is associated with a significant reduction in the incidence of preeclampsia and PIH among primigravidae in a single urban tertiary hospital in Malaysia. Palm oil vitamin E deserves further scrutiny as a potential public health preventive measure against preeclampsia and PIH. A more robust extrinsic validity study is indicated in future in order to consolidate the findings of this study.

## Data Availability Statement

The raw data supporting the conclusions of this article will be made available by the authors, without undue reservation.

## Ethics Statement

The studies involving human participants were reviewed and approved by UKM Research and Ethics Committee. The patients/participants provided their written informed consent to participate in this study.

## Author Contributions

NAA, ZAM, and RS designed the secondary data analysis and wrote the manuscript. NAA and RS analyzed and interpreted the results. All authors contributed to the article and approved the submitted version.

## Conflict of Interest

The authors declare that the research was conducted in the absence of any commercial or financial relationships that could be construed as a potential conflict of interest.

## Abbreviations

ARD: absolute risk difference
FGR: fetal growth restriction
HDP: hypertensive disorders of pregnancy
ISSHP: International Society for the Study of Hypertension in Pregnancy
MYR: Malaysian Ringgit
NNT: numbers needed to treat
PIH: pregnancy induced hypertension
RCT: randomized controlled trial
RR: relative risk
SD: standard deviation
TRF: tocotrienol-rich fraction.
